# ACCELEROMETER-DERIVED PHYSICAL ACTIVITY IN THE NATIONAL HEALTH AND AGING TRENDS STUDY

**DOI:** 10.1093/geroni/igad104.0542

**Published:** 2023-12-21

**Authors:** Jennifer Schrack, Talan Zhang, Amal Wanigatunga, Vadim Zipunnikov, Vicki Freedman

**Affiliations:** Johns Hopkins University, Baltimore, Maryland, United States; Johns Hopkins School of Medicine, Baltimore, Maryland, United States; Johns Hopkins School of Public Health, Baltimore, Maryland, United States; Johns hopkins Bloomberg School of Public Health, Baltimore, Maryland, United States; University of Michigan, Ann Arbor, Michigan, United States

## Abstract

Physical activity (PA) is a modifiable risk factor for disability, chronic conditions, and functional outcomes with aging. Yet the amount of PA in which older adults engage is not well understood, as objective evidence is lacking, particularly among those aged ≥80 years. To address this gap, the National Health and Aging Trends Study (NHATS) implemented wrist-worn accelerometry (Actigraph Centrepoint Insight Watch) in a subset of Round 11 (2021) participants (N=747, 54% women, 40% aged≥80) using a seven-day 24-hour/day protocol. Mean daily PA (total activity counts/day) was examined in those with ≥3 valid wear days by health and demographic factors with analytic survey weights applied. Daily PA averaged 1.67M counts/day, was lower at older ages and varied widely across health and demographic factors, notably: women were more active than men (β=208K), married participants more active than unmarried (β=163K), those with impaired contrast vision were less active than those with normal vision (β=-231K), those with hearing loss were less active than those without hearing loss (β=-233K), and participants with possible or probable dementia were less active than those without dementia (β=-457K) (p<.001 for all). With regards to care and accommodations, those who reported getting help with mobility were less active than those who did not (β=-459K) and those residing in the community were more active than those in (non-nursing home) residential care (β=394K). These data present unique insights into factors that may shape daily PA in older adults. Future work should examine longitudinal trends and potential interaction with the disablement process.

